# Trends Regarding Racial Disparities Among Malnourished Patients With Percutaneous Endoscopic Gastrostomy (PEG) Tubes

**DOI:** 10.7759/cureus.31781

**Published:** 2022-11-22

**Authors:** Ayham Khrais, Mohamed Ismail, Aaron Kahlam, Amjad Shaikh, Sushil Ahlawat

**Affiliations:** 1 Department of Medicine, Rutgers University New Jersey Medical School, Newark, USA; 2 Department of Gastroenterology and Hepatology, Rutgers University New Jersey Medical School, Newark, USA

**Keywords:** race-based differences, feeding nasogastric tube insertion, adult malnutrition, percutaneous endoscopic gastrostomy (peg) feeding, race inequities

## Abstract

Introduction: Percutaneous endoscopic gastrostomy (PEG) is performed in patients who cannot tolerate oral intake and who may require more than 30 days of nutritional support. These patients are at high risk for malnutrition, which itself can contribute to worsening clinical status. Racial disparities regarding access to sources of nutrition have been established. We aim to determine if such racial disparities regarding the diagnosis of malnutrition exist in this high-risk population.

Methods: The National Inpatient Sample (NIS) was queried for patients with International Classification of Diseases, Ninth Revision (ICD-9) diagnoses coding for PEG tube placement with or without a diagnosis of malnutrition. Results were stratified by race. Rates of PEG tube complications were assessed. Categorical and continuous data were assessed via chi-squared and analysis of variance (ANOVA) tests respectively. Binary and multiple logistic regression was used to control for confounders.

Results: Black patients had the highest rates of malnutrition diagnoses, mechanical complications from gastrostomy placement, and the lowest rates of palliative care discussions. Asian or Pacific Islander patients had the highest rates of aspiration pneumonia, gastrointestinal bleeding, the greatest mortality rates, and the longest hospital stays.

Discussion: Racial minorities had worse outcomes while Caucasians had shorter hospital stays and lower complication rates. Such disparities can be multifactorial in etiology, with lack of nutritional access, poor doctor-patient communication, and differential rates of insurance coverage contributing to poorer outcomes among racial minorities. More change is required to promote equity when managing patients with end-of-life diseases necessitating methods of nutritional support.

## Introduction

Percutaneous endoscopic gastrostomy (PEG) is a procedure involving the insertion of a gastric feeding tube via endoscopy [1,2]. PEGs are indicated in patients who are either unable to tolerate or complete adequate oral intake and who therefore cannot deliver food boluses from the oral cavity to the stomach [2]. While alternate methods of nutritional delivery (i.e., nasogastric tubes) may be used for feeding, such mechanisms are only short-term (less than 30 days) solutions due to a variety of associated complications including aspiration and bleeding [1,3]. PEGs are reserved for patients with feeding difficulties who will require more than 30 days of nutritional support and who would therefore be at significant risk for malnutrition if such delivery systems were not put in place [1,2]. Examples of such patients include those with neurologic and cerebrovascular disease (i.e., stroke-induced dysphagia, multiple sclerosis, cerebral palsy), patients with head and neck cancers, patients with esophageal cancer, and patients who have suffered severe burns [1,4]. PEGs can be placed for non-nutritional indications as well, including gastric decompression in the case of obstructive intra-abdominal malignancies [1,5]. On the other hand, they are not indicated in patients with a poor prognosis, including patients of advanced age. Reported complication rates vary widely from 16% to 88% [6]. Some are associated with endoscopic procedures, including aspiration (0.06%), gastrointestinal bleeding (1%), and perforation of the alimentary canal (0.04%) [5,6]. Other complications of PEG placement involve damage to intra-abdominal vasculature, resulting in intra- or retroperitoneal bleeding and damage to various intra-abdominal organs [5]. Complications of PEG use include ostomy infection (18%), gastrointestinal bleeding (2.5%), aspiration pneumonia, and “buried bumper syndrome” (1.9%), which describes the migration of the internal bumper of the feeding tube into the stoma with subsequent growth of the gastric mucosa over the displaced internal bumper [5-9].

Gastric feeding tubes are placed to maintain proper nutrition in at-risk patients, with evidence suggesting that placement results in weight gain in malnourished patients, however, other literature reviews suggest that in patients with dementia, PEG placement does not ameliorate poor nutritional status or survival rates [5,8-10].

Malnutrition, a major indication for PEG placement, depicts a shortage of nutrients and minerals required for the maintenance of homeostasis [11]. This depletion of energy sources can be due to malignancy, decreased oral intake, or impaired absorption [11]. This dearth of energy stores results in muscle wasting, immunocompromise, and worsening inpatient outcomes including mortality [12]. Therefore, adequately managing malnutrition via interventions such as feeding tube placement is critical in improving outcomes. However, rates of PEG placement, and its risks, have been shown to differ among patients of different races and household incomes who have suffered a cerebrovascular accident, a major indication for the procedure [13,14]. Our goal is to determine if similar race-based differences exist in the identification of malnutrition in patients who have undergone PEG placement. Furthermore, we aim to characterize race-based differences in procedural complications, mortality, palliative care discussions, and social determinants of health in patients with PEGs.

## Materials and methods

Data source

Sample data ranging from 2001 to 2013 were obtained from the National Inpatient Sample (NIS). Developed by the Agency for Healthcare Research and Quality, the NIS is the largest public all-payer inpatient database containing information on more than 7 million hospital stays in the United States. The NIS database contains no patient or hospital identifiers and provides a nationally representative set of data reflecting 20% of all discharges from hospitals within the United States. International Classification of Diseases, Ninth Revision, Clinical Modification (ICD-9 CM) codes were used to identify cases of interest.

Study design

Inclusion criteria consisted of patients 18 years of age or older with an ICD-9 code for PEG tube placement. Patients were stratified based on an ICD-9 diagnostic code for malnutrition. Therefore, two groups were formed, those with a PEG tube and a diagnosis of malnutrition, and those with a PEG tube without a diagnosis of malnutrition (Table 1). Patient race (White, Black, Hispanic, Asian or Pacific Islander, Native American) was identified and compared to other patient demographics (age, sex at birth), social determinants of health (primary payer, median household income), and outcomes including mortality, length of stay, hospital costs, and PEG tube complications (aspiration pneumonia, gastrointestinal bleeding, mechanical PEG complications). Further comparisons involving palliative care discussions and post-discharge disposition were made as well.

**Table 1:** ICD-9 Codes and Associated Diagnoses. ICD-9: International Classification of Diseases, Ninth Revision

Diagnosis	ICD-9 Code
Percutaneous endoscopic gastrostomy (PEG) tube	43.11
Malnutrition	263.0, 263.1, 263.8, 263.9
Aspiration pneumonia	507.0
Encounter for palliative care	V66.7
Mechanical PEG complications	536.40, 536.42
Gastrointestinal bleeding	578.0, 578.1, 578.9

Statistical analyses

Chi-squared and analysis of variance (ANOVA) tests were conducted to assess categorical and continuous variables respectively. Binomial and multiple logistic regression analyses were performed to adjust data for the following confounding variables: age, sex at birth, cardiac arrhythmias, chronic obstructive pulmonary disease, congestive heart failure, diabetes mellitus, human immunodeficiency virus, hypertension, peripheral artery disease, peptic ulcer disease, renal failure, and paralysis. Microsoft Excel was utilized to generate a graphic representation of yearly trends in the prevalence of malnourished patients with PEG tubes, divided by race.

## Results

A total of 390,275 patients from 2001 to 2013 had PEG tubes, 113,032 of whom were diagnosed with malnutrition (Table 1). Most of the patients with a diagnosis of malnutrition who had PEG tubes placed were White (n=250,752), followed by Black (n=76,438), Hispanic (n=37,261), Asian or Pacific Islander (n=12,509), and finally Native American (n=1,645). Asian or Pacific Islanders were, on average, older (73.71 years; p<0.05), followed by White patients (71.77 years); Native Americans were, on average, the youngest at 66.65 years old (Table 2). Most patients among all racial groups were male, except Black patients, the majority of whom were female (54.5%; p<0.05). Medicare was the most common primary payer among all racial groups, however, White patients had the highest rates of utilization of private insurance (16.2%; p<0.05), as well as the lowest rates of self-pay (1.8%). Hispanics (3.9%) and Native Americans (3.5%) had the highest rates of self-pay as the primary insurance payer (Table 2).

In terms of median household income, most Asians or Pacific Islanders were within the 4th quartile (62%; p<0.05), followed by White patients (49.3%) and then Native Americans (43.7%). Black patients were the fewest within the 4th quartile (30.9%), and the majority of Black patients were within the 2nd quartile for median household income (29.7%).

Regarding discharge disposition (Table 2), the majority of patients within all racial groups were discharged to a nursing facility. Black patients had the lowest rates of discharge to home healthcare (11.8%; p<0.05). Native Americans had the highest rates of out-of-hospital deaths (11.9%).

**Table 2:** Patient Demographics. n: sample size

		Race					p-value
		White (n=250,752)	Black (n=76,438)	Hispanic (n=37,261)	Asian or Pacific Islander (n=12,509)	Native American (n=1,645)	
Age (years)		71.77	69.44	69.61	73.71	66.65	<0.05
Sex at birth	Male n (%)	133,327 (53.2%)	34,754 (45.5%)	19,895 (53.4%)	6,519 (52.1%)	865 (52.6%)	<0.05
	Female n (%)	117,348 (46.8%)	41,665 (54.5%)	17,361 (46.6%)	5,989 (47.9%)	780 (47.4%)	
Primary payer	Medicare n (%)	184,726 (73.8%)	52,998 (69.5%)	23,819 (64.0%)	8,303 (66.4%)	1,009 (61.6%)	<0.05
	Medicaid n (%)	16,040 (6.4%)	11,132 (14.6%)	6,637 (17.8%)	2,131 (17.0%)	280 (17.1%)	
	Private insurance n (%)	40,507 (16.2%)	8,719 (11.4%)	4,345 (11.7%)	1,643 (13.1%)	215 (13.1%)	
	Self-pay n (%)	4,418 (1.8%)	2,032 (2.7%)	1,456 (3.9%)	251 (2.0%)	58 (3.5%)	
	No charge n (%)	461 (0.2%)	239 (0.3%)	174 (0.5%)	16 (0.1%)	7 (0.4%)	
Median household income	1^st^ Quartile n (%)	1,295 (3.6%)	1,841 (17.2%)	832 (18.2%)	52 (3.2%)	12 (11.7%)	<0.05
	2^nd^ Quartile n (%)	7,386 (20.6%)	3,188 (29.7%)	1,270 (27.9%)	181 (11.3%)	24 (23.3%)	
	3^rd^ Quartile n (%)	9,473 (26.5%)	2,840 (26.5%)	1,047 (23.0%)	378 (23.5%)	22 (21.4%)	
	4^th^ Quartile n (%)	17,626 (49.3%)	2,854 (26.6%)	1,411 (30.9%)	997 (62.0%)	45 (43.7%)	
Discharge disposition	Routine n (%)	13,126 (10.4%)	3,962 (10.4%)	2,883 (15.1%)	760 (12.5%)	88 (13.1%)	<0.05
	Short-term hospital n (%)	3,430 (2.7%)	912 (2.4%)	704 (3.7%)	223 (3.7%)	17 (2.5%)	
	Nursing facility n (%)	79,636 (63.0%)	24,567 (64.3%)	10,907 (57.2%)	3,599 (59.1%)	403 (60.1%)	
	Home healthcare n (%)	15,582 (12.3%)	4,509 (11.8%)	2,426 (12.7%)	744 (12.2%)	82 (12.2%)	
	Out-of-hospital death n (%)	14,289 (11.3%)	4,180 (10.9%)	2,050 (10.8%)	713 (11.7%)	80 (11.9%)	

Malnutrition was most diagnosed in Black patients (31.5%), followed by White patients (28.9%), Native Americans (28.8%), Asian or Pacific Islanders (27.7%), and then Hispanics (25.5%) (Table 3). In terms of PEG complications, Asian or Pacific Islander patients had the highest rates of aspiration pneumonia (30.6%; p<0.05), while Black patients had the lowest rates (24.3%) (Table 3). Asian or Pacific Islander patients had the highest rates of gastrointestinal bleeding (5.1%; p<0.05), followed by Black patients (4.1%). Black patients had the highest rates of mechanical PEG complications (2.5%; p<0.05), while White patients had the lowest rates (1.5%). Black patients had the lowest rates of palliative care discussions (2.2%; p<0.05), while Asian or Pacific Islander patients had the highest rates at 3.1%. Mortality was greatest in Asian or Pacific Islander patients (10.1%; p<0.05), followed by White patients (9.7%), then Hispanic patients (9.6%). Length of stay was greatest in Asian or Pacific Islander patients (22.88 days; SD 25.193±0.225). White patients had the lowest length of stay (20.29 days; SD 19.905±0.04).

**Table 3:** Rates of PEG Complications and Primary Inpatient Outcomes. PEG: percutaneous endoscopic gastrostomy, n: sample size, USD: United States dollar, SD: standard deviation, SE: standard error

Outcomes and PEG Complications		Race					p-value
		White	Black	Hispanic	Asian or Pacific Islander	Native American	
Malnutrition n (%)	Yes	72,400 (28.9%)	24,079 (31.5%)	9,489 (25.5%)	3,463 (27.7%)	474 (28.8%)	<0.05
	No	178,352 (71.1%)	52,359 (68.5%)	27,772 (74.5%)	9,046 (72.3%)	1,171 (71.2%)	
Aspiration pneumonia n (%)	Yes	68,132 (27.2%)	14,956 (19.6%)	9,038 (24.3%)	3,866 (30.6%)	411 (25.0%)	<0.05
	No	182,620 (72.8%)	61,482 (80.4%)	28,223 (75.7%)	8,643 (69.1%)	1,234 (75.0%)	
Gastrointestinal bleeding n (%)	Yes	9,293 (3.7%)	3,166 (4.1%)	1,388 (3.7%)	641 (5.1%)	63 (3.8%)	<0.05
	No	241,459 (96.3%)	73,272 (95.9%)	35,873 (96.3%)	11,868 (94.9%)	1,582 (96.2%)	
Mechanical PEG complication n (%)	Yes	3,667 (1.5%)	1,921 (2.5%)	771 (2.1%)	260 (2.1%)	34 (2.1%)	<0.05
	No	247,085 (98.5%)	74,517 (97.5%)	36,490 (97.9%)	12,249 (97.9%)	1,611 (97.9%)	
Encounter for palliative care n (%)	Yes	6,268 (2.5%)	1,705 (2.2%)	784 (2.1%)	391 (3.1%)	38 (2.3%)	<0.05
	No	244,484 (97.5%)	74,733 (97.8%)	36,477 (97.9%)	12,118 (96.9%)	1,607 (97.7%)	
Mortality n (%)		24,311 (9.7%)	7,194 (9.4%)	3,573 (9.6%)	1,258 (10.1%)	136 (8.3%)	<0.05
Length of stay (days) n (SD±SE)		20.29 (19.905±0.04)	21.81 (22.461±0.081)	22.20 (23.105±0.12)	22.88 (25.193±0.225)	22.55 (23.532±0.58)	<0.05
Hospital costs (USD) n (SD±SE)		139,792.08 (180,085.46±362.377)	135,452.77 (183,191.047±668.46)	182,722.77 (230,081.3±1,204.515)	198,937.7 (260,106.502±2,377.609)	163,335.9 (208,689.727±5,176.955)	<0.05

After binomial and multiple regression analyses, most outcomes including social determinants of health (primary payer, median household income), mortality, length of stay, hospital costs, and PEG tube complications (aspiration pneumonia, gastrointestinal bleeding, mechanical PEG complications) were statistically significant (Table 4). In terms of the primary payer, after adjustment for confounding variables, all correlations with race were statistically significant with the exception of that between medicare and both Hispanic and Asian or Pacific Islander patients, private insurance and Black patients, self-pay and Black, Hispanic, and Asian or Pacific Islander patients, as well as no pay and Black, Hispanic, Asian or Pacific Islander, and Native American patients (Table 4). Discharge dispositions were statistically significant when assessing those of Hispanic and Asian or Pacific Islander patients (Table 4).

**Table 4:** Adjusted Odds Ratios and p-Values of Inpatient Outcomes Generated from Binomial and Multiple Logistic Regression Analyses. PEG: percutaneous endoscopic gastrostomy, OR: odds ratio, CI: 95% confidence interval, n: sample size

		Race				
		White OR (CI); p-value	Black OR (CI); p-value	Hispanic OR (CI); p-value	Asian or Pacific Islander OR (CI); p-value	Native American OR (CI); p-value
Malnutrition		1.146 (1.125-1.166); p<0.05	0.847 (0.826-0.869); p<0.05	0.969 (0.931-1.009); p=0.132	0.999 (0.897-1.112); p=0.979	0.916 (0.879-0.956); p<0.05
Mortality		0.956 (0.929-0.984); p<0.05	0.998 (0.961-1.036); p=0.925	1.090 (1.025-1.158); p=0.05	0.837 (0.7-1); p<0.05	1.115 (1.049-1.186); p<0.05
Aspiration pneumonia		0.675 (0.661-0.689); p<0.05	0.878 (0.856-0.901); p<0.05	1.239 (1.191-1.289); p<0.05	0.907 (0.810-1.015); p=0.088	0.915 (0.877-0.955); p<0.05
Gastrointestinal bleeding		1.122 (1.076-1.170); p<0.05	1.012 (0.955-1.072); p=0.689	1.445 (1.330-1.569); p<0.05	1.032 (0.801-1.329); p=0.808	1.203 (1.098-1.318); p<0.05
Mechanical PEG complications		1.8 (1.701-0.906); p<0.05	1.445 (1.336-1.564); p<0.05	1.48 (1.303-1.681); p<0.05	1.436 (1.02-2.02); p<0.05	1.265 (1.1-1.455); p<0.05
Encounter for palliative care		0.835 (0.79-0.882); p<0.05	0.814 (0.755-0.878); p<0.05	1.208 (1.089-1.341); p<0.05	0.9 (0.651-1.243); p=0.521	0.976 (0.866-1.099); p=0.683
Primary payer	Medicare	1.491 (1.303-1.707); p<0.05	1.708 (1.478-1.974); p<0.05	0.938 (0.808-1.09); p=0.407	1.099 (0.895-1.348); p=0.369	0.645 (0.484-0.858); p<0.05
	Medicaid	0.543 (0.473-0.625); p<0.05	1.27 (1.094-1.474); p<0.05	1.183 (1.014-1.379); p<0.05	2.029 (1.646-2.502); p<0.05	0.547 (0.407-0.735); p<0.05
	Private insurance	1.288 (1.122-1.479); p<0.05	0.98 (0.845-1.137); p=0.789	0.729 (0.625-0.85); p<0.05	1.317 (1.068-1.625); p<0.05	0.408 (0.302-0.551); p<0.05
	Self-pay	0.539 (0.459-0.634); p<0.05	0.88 (0.741-1.045); p=0.145	0.916 (0.767-1.095); p=0.334	0.849 (0.661-1.092); p=0.203	0.411 (0.281-0.602); p<0.05
	No charge	0.603 (0.429-0.846); p<0.05	1.114 (0.782-1.587); p=0.551	1.193 (0.829-1.717); p=0.341	0.572 (0.312-1.051); p=0.072	0.536 (0.231-1.244); p=0.147
Discharge disposition	Routine	1.094 (0.607-1.97); p=0.765	1.262 (0.668-2.383); p=0.473	0.497 (0.27-0.914); p<0.05	0.184 (0.099-0.343); p<0.05	0.665 (0.185-2.396); p=0.533
	Short-term hospital	1.248 (0.686-2.271); p=0.468	1.329 (0.696-2.536); p=0.389	0.43 (0.231-0.801); p<0.05	0.244 (0.129-0.46); p<0.05	1.104 (0.302-4.034); p=0.881
	Nursing facility	1.088 (0.608-1.95); p=0.776	1.533 (0.816-2.878); p=0.184	0.36 (0.197-0.658); p<0.05	0.147 (0.079-0.271); p<0.05	0.598 (0.168-2.122); p=0.426
	Home healthcare	1.345 (0.748-2.419); p=0.3 22	1.565 (0.831-2.95); p=0.166	0.441 (0.24-0.811; p<0.05	0.201 (0.108-0.372); p<0.05	0.579 (0.161-2.081); p=0.403
	Out-of-hospital death	0.922 (0.512-1.66); p=0.786	1.205 (0.639-2.275); p=0.564	0.34 (0.185-0.625); p<0.05	0.142 (0.076-0.264); p<0.05	0.379 (0.104-1.382); p=0.142

**Figure 1:**
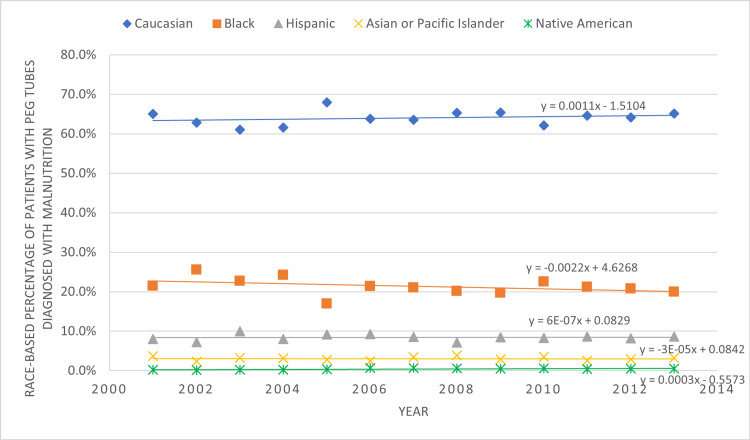
Yearly Trends in Race-Based Prevalence of Malnutrition Among Patients with PEG Tubes with p<0.05. PEG: Percutaneous endoscopic gastrostomy

From 2001 to 2013, racial proportions of patients with PEG tubes diagnosed with malnutrition remained mostly consistent, with rates of Black patients decreasing steadily with time, while those of White patients were increasing at a slow rate (Figure 1). In effect, racial differences did not change significantly over a period of 12 years.

## Discussion

The results of this study revealed significant racial disparities not only in access to healthcare, but also in rates of PEG tube complications. We found disproportionately greater access to healthcare in Caucasian patients by means of the greatest ability to access private insurance and the lowest rates of self-pay. Similar trends have been shown in other research endeavors, whereby Caucasian patients have higher rates of utilization of private insurance and lower rates of self-pay [15,16]. On the other hand, we found that patients of racial minorities had higher rates of self-pay. Lack of insurance in minorities can lead to poorer health outcomes, as these patients will have decreased access to healthcare professionals, screening, diagnostic tests, and treatment options [16]. These patients are therefore at higher risk for poor outcomes, including procedural complication rates, mortality, and length of stay compared to those with insurance.

We found that Black patients had the highest rates of malnutrition diagnoses. Previous studies have shown an elevated prevalence of poor nutrition in Black patients versus Caucasians [17]. Black patients had poorer nutritional intake (including fruits and vegetables), and older Black patients were at greater risk for malnutrition than their Caucasian counterparts [14,15,17]. Healthy food items are generally more expensive than less healthy choices, thereby limiting their availability to low-income populations [18]. Food deserts are described as regions with both poor access to supermarkets and low average income [18,19]. Patients without access to supermarkets are unable to cook their own food and are therefore forced to eat food prepared by fast food restaurants and other such sources, which have been shown to lack adequate nutrition [19]. Black patients have been shown to be at higher risk of living in food deserts and of not eating homemade food, in effect placing them at higher risk for poor nutritional intake [19]. Therefore, pre-existing poor nutritional status among Black patients could have contributed to higher rates of malnutrition among this population.

In terms of outcomes, Caucasians had lower rates of PEG complications, hospital costs, and lengths of stay compared to members of other races. There is substantial evidence regarding race-based differences in post-procedural outcomes, including appendectomy and bariatric surgery [20,21]. Black and Hispanic patients have been shown to have higher rates of post-procedural complications, mortality, lengths of stay, and rates of hospital re-admission [20,21]. These differences can be a result of poor doctor-patient communication and socioeconomic factors prevalent in each racial subgroup [22,23]. Research suggests that if a physician and their patient are of different races, then they may have poorer communication between them [22]. Therefore, pre- and post-procedural instructions as well as conveyance of procedural complications may be inadequate among patients within the racial minority leading to poorer patient compliance [22]. Furthermore, socioeconomic factors including income, education, and medical insurance can significantly impact patients’ health literacy, ability to afford medications, and compliance with physician appointments [16,22]. Racial minorities, including African Americans and Hispanics, have been shown to have an increased prevalence of insufficient insurance coverage and poor health literacy [16,24]. This can impact their ability to understand their disease process and treatment options, which translates into reduced compliance with medications and pre- and post-procedural instructions, thereby leading to increased complication rates, mortality, and re-admission rates [20-22].

Out of all studied minority groups, Black patients had the lowest rates of palliative care discussions, while Caucasian patients had the second highest rate of palliative care discussions. Studies show contrasting race-based utilization of palliative services exists among patients with varying terminal illnesses. Research regarding patients with terminal gynecologic malignancies, including uterine and ovarian cancer, has demonstrated lower rates of palliative care utilization in Black patients and higher rates in Caucasians [25]. On the other hand, other studies analyzing Black patients with terminal malignancies have demonstrated higher rates of palliative care discussions in this population when compared to Caucasians [26,27]. Further research is needed to adequately identify race-based differences in the utilization of palliative care services, for these services have been shown to improve the quality of life in patients with end-stage illnesses [26]. Therefore, this service must be provided to all individuals, when indicated, on a fair basis regardless of race or ethnicity.

Our study is limited by the amount of information housed within the NIS database and does not include the severity of malnutrition or of other comorbidities that could be influencing complication rates, lengths of stay, hospital costs, and mortality. The NIS database also does not include the time physicians spent counseling patients and time spent with palliative care discussions. Furthermore, the database identifies medical conditions via the utilization of ICD-9 diagnostic codes. If a hospitalized patient with malnutrition is not provided with an ICD-9 code for that diagnosis, then the sample we studied may underestimate the prevalence of malnutrition among the population. Such underestimation can affect observed rates of PEG complications as well as racial discrepancies between them and other measured outcomes.

## Conclusions

Racial discrepancies in procedural complication rates, hospital outcomes, and palliative care utilization have been previously established. Here, we reveal that similar discrepancies exist among patients with PEG tubes. Caucasian patients overall had lower complication rates and better outcomes, while Black patients had higher rates of malnutrition, worse outcomes, and lower rates of palliative care discussions. These results suggest that, within the past few decades, racial disparities in healthcare have affected patient management and hospital courses, with minorities suffering from poorer outcomes overall. Since trends in the diagnosis of malnutrition among patients of different races have remained consistent within the past decade, more change is needed in order to promote equity in the practice of medicine, especially when managing impressible patient populations such as those with terminal illnesses or dysphagia requiring PEG tubes and end-of-life discussions.
